# Barriers and Facilitators to Breast and Cervical Cancer Screenings for Hispanic Women in a Rural State

**DOI:** 10.1007/s40615-024-02037-6

**Published:** 2024-06-07

**Authors:** Sally Moyce, David Claudio, Elizabeth Aghbashian, Kelly Keenan, Danika Lee Comey, Genesis Chavez-Reyes

**Affiliations:** 1 College of Nursing, Montana State University, Bozeman, MT USA; 2 Department of Mechanical and Industrial Engineering, University of Massachusetts Lowell, Lowell, MA USA; 3 Gallatin City-County Health Department, Gallatin County, Bozeman, MT USA

**Keywords:** Breast and cervical cancer screening, Hispanic, Community-based participatory research, Qualitative research, Systematic process improvement approach

## Abstract

**Introduction:**

The Centers for Disease Control and Prevention (CDC) and the Division of Cancer Prevention of Control administer the National Breast and Cervical Cancer Early Detection Program (NBCCEDP), designed to increase early detection of cancers among low-income uninsured and underinsured women. However, rates of cancer diagnosis and survivorship differ among women of different ethnicities. We investigated two questions: 1) what are the potential barriers and facilitators for women to complete recommended breast and cervical cancer screenings, and 2) are the barriers and facilitators different for Hispanic women when compared to non-Hispanic White women?

**Methods:**

We used a community-based participatory research approach and mixed methods: qualitative interviews with women enrolled in the program and a systematic process improvement approach to identify root causes of completing or not completing screenings. We conducted semi-structured interviews in English (*n* = 11) and Spanish (*n* = 9) and analyzed responses using fishbone diagrams.

**Results:**

We recruited 20 participants in four categories: (a) non-Hispanic White women who completed screenings (*n* = 9), (b) non-Hispanic White women who did not complete screenings (*n* = 2), (c) Hispanic women who completed screenings (*n* = 7), and (d) Hispanic women who did not complete screenings (*n* = 2). Among all women, facilitators included assistance from program staff with appointments and reminders. Hispanic women reported barriers including language difficulties and confusion about the program. Non-Hispanic White women identified barriers as confusion about the role of insurance.

**Conclusions:**

We found that there are differences in barriers and facilitators for non-Hispanic White women and Hispanic women due to language, the role of insurance, and the level of trust in the program. Reasons for not completing screenings for Hispanic women were structural and systemic in nature; reasons for non-Hispanic White women were based on personal choices.

## Introduction

The Centers for Disease Control and Prevention (CDC) and the Division of Cancer Prevention of Control administer the National Breast and Cervical Cancer Early Detection Program (NBCCEDP), designed to increase early detection of cancers among low-income uninsured and underinsured women [1]. Working through agreements with local health departments, the program has improved access to cancer screenings for many women, especially minoritized women in its more than 30 years of operation [2, 3].

In Southwest Montana, the program is administered in three counties through the Gallatin City-County Health Department. The health department provides vouchers for free screenings to eligible women through their primary care providers and health outreach efforts [4]. According to our analysis of historical data provided by the Gallatin City-County Health Department, the program has provided access to screening services for 200 women annually since 2016, an estimated 8% of eligible women. In the last 5 years, 20 women have been diagnosed with breast cancer and 13 diagnosed with cervical cancer through services funded by the program. Cancer is the second leading cause of death of Montana residents, and approximately 5600 new cases are diagnosed each year [4]. Women are at risk of developing breast cancer, and it is the most frequently diagnosed malignancy in women [5]. The incidence of breast cancer in Montana is 13.6% and accounts for 7% of deaths [6]. The incidence of cervical cancer is 7.9% [7]. Breast and cervical cancer are curable in the majority of women, especially when it is caught early [8]. However, rates of cancer diagnosis and survivorship differ among women of different ethnicities. Compared to non-Hispanic White women, Hispanic women are often diagnosed with more advanced disease, have lower rates of 5-year survival, and have lower quality of life [9]. Hispanic women also have lower levels of knowledge about their breast cancer and report lower levels of satisfaction with the quality of their cancer care [10].

While the proportion of Latinos in southwest Montana is below 5%, [11] Montana is a new immigrant destination with a growing number of Latino residents [12]. Despite the recent growth, the health needs of the Latino population are largely unmet. Only 20% of the Latino population in this area report having health insurance compared to 87% of the non-Hispanic White population [13]. Persons who lack health insurance or a regular source of care are more likely to experience barriers to preventive screening [14]. Latinos experience disproportionately higher barriers to accessing screening than non-Hispanic Whites for a variety of reasons [15]. Cultural differences are a large part of this disparity, and Latinos tend to not engage in the care continuum due to a lack of providers who speak their language, understand their culture, and are geographically accessible to them [16–18]. This is exacerbated in a rural setting where the geography often prevents residents from accessing needed medical care [19, 20].

To investigate potential disparities in accessing preventive screenings for Hispanic women in the Montana, we undertook a study to investigate the following two questions: 1) what are the potential barriers and facilitators for women to complete recommended breast and cervical cancer screenings, and 2) are the barriers and facilitators different for Hispanic women when compared to non-Hispanic White women?

## Methods

We used a mixed methods approach to answer our research questions: qualitative interviews with women and a systematic process improvement approach. Systematic process improvement is an engineering approach that has been employed in studies designed to improve healthcare quality. We chose it because of its step-by-step analysis of how processes contribute to outcomes [21, 22]. We used a retroactive case comparison design to determine potential differences in barriers and facilitators between women who completed the program and women who did not complete the program and differences based on ethnicity.

### Community Advisory Board

We engaged a Community Advisory Board (CAB) of Hispanic women to advise our work. The women were known to the research team through various interactions and were selected because of their role in the community. Three of the women had completed the NBCCEDP program; one had not. Three of the women spoke Spanish; one spoke only English. We met with the CAB four times during the study. First, we met to get input on the research question and to determine the best way to answer the question. They suggested interviewing women who had enrolled in NBCCEDP. In a second meeting, the CAB developed the interview guide. We also asked the director of the local cancer support center to review the interview guide. The interview guide was pilot tested on three of the CAB members over the phone and further refined based on feedback from the CAB members.

The CAB assisted the team in contacting and recruiting participants to the study through their personal and professional connections. Following the qualitative interviews, we asked the CAB to complete their own fishbone diagram based on their understanding of barriers and facilitators to completing screenings. In a fourth meeting, we conducted a concept mapping exercise [23] with the CAB to examine final results and to brainstorm potential solutions (reported elsewhere).

All meetings with the CAB lasted approximately 90 min. We provided a meal and compensated members $100 for their time.

### Study Participants

We obtained a list of all women in the three counties who had enrolled or been contacted about the NBCCED in 2021 and 2022 from records submitted to the state by program administrators. We chose these years because they were recent enough to allow women to recall their experiences during qualitative interviews. We divided the list of women into four categories based on their enrollment records: (1) self-identified non-Hispanic White woman who completed all recommended NBCCEDP screenings (*n* = 37); (2) self-identified non-Hispanic, White women who did not complete NBCCEDP screenings (*n* = 10); (3) self-identified Hispanic woman who completed all recommended NBCCEDP screenings (*n* = 35); and (4) self-identified Hispanic women who did not complete NBCCEDP screenings (*n* = 10). Using the phone of the NBCCEDP administrator (to provide a familiar phone number), all women on the list were texted with a brief description of the study.

Participants were contacted a maximum of three times via text message and invited to engage in a semi-structured interview over the phone regarding their experiences with the NBCCEDP. Interested women were contacted by a bilingual research assistant to schedule the interview. Prior to beginning the interview, the research assistant explained the purpose of the study and its objectives and obtained verbal informed consent for participating in the interviews and for being audio-recorded. At the end of each interview, participants were mailed a written copy of the consent form containing investigators’ contact information and a gift card as compensation for their time and experiences. We utilized a tracking sheet to monitor the interview competition process and to ensure that the protocol was followed as designed.

### Interviews

Interviews were conducted over the phone by two research assistants—one native English speaker and one native Spanish speaker—in the fall of 2022. Interviews were scheduled for a time convenient to participants and women who missed the interview appointments were contacted three times to reschedule. After three attempts, we asked one of the CAB members to attempt to contact participants to reschedule appointments. Interviews lasted approximately 45 min; participants were given the opportunity to contact us after the interviews if they wanted to add additional information. Calls were audio recorded and transcribed in their original language using NVivo qualitative software. Spanish-language interviews (*n* = 9) were then translated by our team. All transcripts were analyzed in English.

### Analysis

We adopted a systematic analysis approach, using engineering tools from process improvement [24]. Rather than conducting a thematic analysis which categorizes respondent quotations under themes that emerge from the data, we selected this approach because we wanted to understand the barriers and facilitators to completing screenings, and the engineering tools allow investigators to systematically identify root causes of an event. We used a fishbone diagram, or Ishikawa diagram, as a framework for identifying the barriers and facilitators to completing recommended screening once enrolled in the NBCCEDP. The fishbone diagram is a tool used to analyze an event and determine the contributors to the event. It gets its name from its shape, where the head of the fish represents the event and the bones represent contributing factors [25, 26]. We labeled the head of the fish according to the group who was interviewed (e.g., Hispanic women who completed all NBCCEDP screenings). We used a deductive approach and selected the categories of the analysis a priori and labeled the bones according to general categories of potential contributors, based on published categories in the literature [27, 28] and adding payment to include financial concerns we hypothesized to be a contributor: (1) *providers* included program administrator, referral sources, imaging personnel, hospital personnel, etc.; (2) *people* included the patients themselves; (3) *process* included factors related to enrolling in the program and completing the screenings; (4) *place* included factors about the referral agencies and the hospital where mammograms were offered; (5) *payment* included factors related to billing and financial responsibilities; and (6) *policy* included factors related to the program itself and its administration.

All members of the research team (three principal investigators and two graduate research assistants) read through all transcripts of the interviews. All members of the team identified common themes that fit into each of the fishbone categories. Each member of the team completed the diagrams independently. The team then met to discuss each participant group and create a complete fishbone diagram for each of the four groups. Differences between investigators in categorization were resolved with discussion and consensus.

Following the data coding by the research team, the CAB was asked to complete their own fishbone diagram related to potential factors that could facilitate or deter a woman from completing recommended screenings. We did not ask the CAB to examine factors based on a particular participant category, rather for women in general who were enrolled in the process. We used this to check our work and to add trustworthiness to the approach. This discussion was led by an external, trained facilitator to mitigate bias.

### Ethical Review

All study procedures were reviewed and approved by the Institutional Review Board at Montana State University.

## Results

We recruited 20 participants to the study in the following categories: (1) self-identified non-Hispanic White woman who completed all recommended screenings (*n* = 9); (2) self-identified non-Hispanic White women who did not complete screenings (*n* = 2); (3) self-identified Hispanic women who completed all recommended screenings (*n* = 7); and (4) self-identified Hispanic women who did not complete screenings (*n* = 2).

### Non-Hispanic White Women Who Completed Screenings

These women identified positive interactions with providers, including reminder calls from the program administrators and clear organization at the providers’ offices. They expressed satisfaction with the screening process, saying it was simple and quick. One woman commented:

*It was good. It was pretty easy. I mean, you know, I had a couple of different appointments . . . But it was straightforward.*

They also noted that access to the screening program was straightforward through a mobile health promotion unit sponsored by a local health system. Negative reactions involved confusion over the role of health insurance and confusion when they received bills for a program they understood to be free. For example, one woman said:

*And I so I had insurance also. And so it was like a little confusing because they don’t cover it like if your insurance covers it, which was like a little confusing for me. But the [program administrator] called me and explained it all.*

**Fig. 1:**
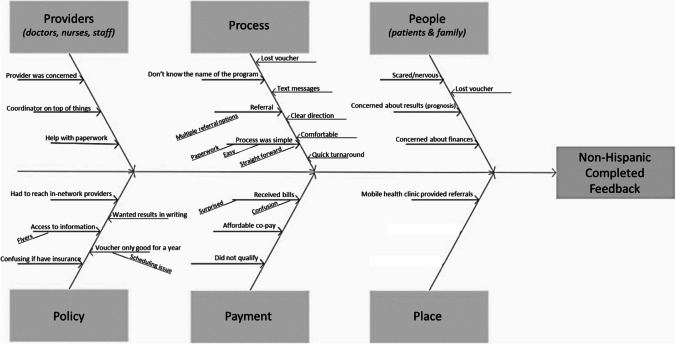
Fishbone diagram constructed from non-Hispanic White women who completed screenings

These women expressed feeling concern and nervousness around the procedure because of a potential positive cancer diagnosis (Fig. 1).

### Non-Hispanic White Women Who Did Not Complete Screenings

Similar to the women who completed screenings, these women expressed satisfaction with the providers and the reminder texts. These women also expressed confusion about the role of insurance and mentioned a co-payment that surprised them. The rationales provided for not completing the program included procrastination, lack of time due to personal life events, and not prioritizing their screenings. For example, one respondent said:

*You know, I’m going to be very honest. Everybody has been wonderful. I have just had a year that I have been a procrastinator. And so I’m going to own it and admit it. I’ve just behind on all my things that I need to be have done.*

**Fig. 2:**
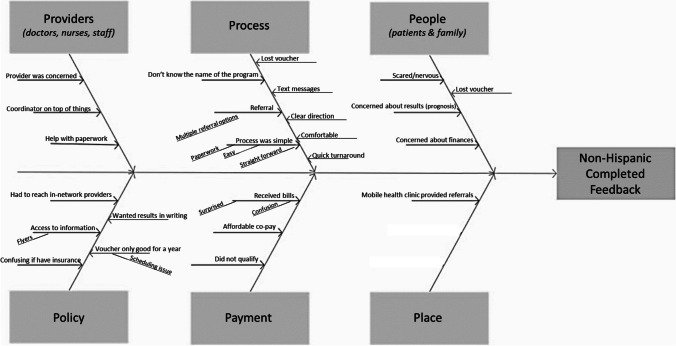
Fishbone diagram constructed from non-Hispanic White women who did not completed screenings

Another respondent said that she was experiencing depression following the death of a spouse, which prevented her from attending to her personal health (Fig. 2).

### Hispanic Women Who Completed Screenings

Like the previous two groups, these women were satisfied and thankful for the role of providers, citing the help of bilingual providers and the bilingual program coordinator. They reported simplicity in the process and told us their provider’s offices scheduled the mammogram appointments for them. One woman told us:

*I remember that [the program administrator] contacted me and very kindly told me not to worry. Also at the clinic, they helped me to make the appointment . . . and they supported me a lot. They helped me.*

They were interested to learn about their own health, and some women told us they provided their own interpreters for visits. Negative feedback on the process included confusion around accessing screenings and reasons for getting screened. The women mentioned a lack of Spanish resources repeatedly, telling us that they received calls in English, results in English, and only encountered English speakers at the mammogram provider. One woman said it was difficult for her to make an appointment because of the language barrier:

*When you call [the imaging center] they put on this recording in English. And sometimes you don’t understand. And if I leave a message, I don’t think they understand me, So I think that is why they don’t call us back.*

**Fig. 3:**
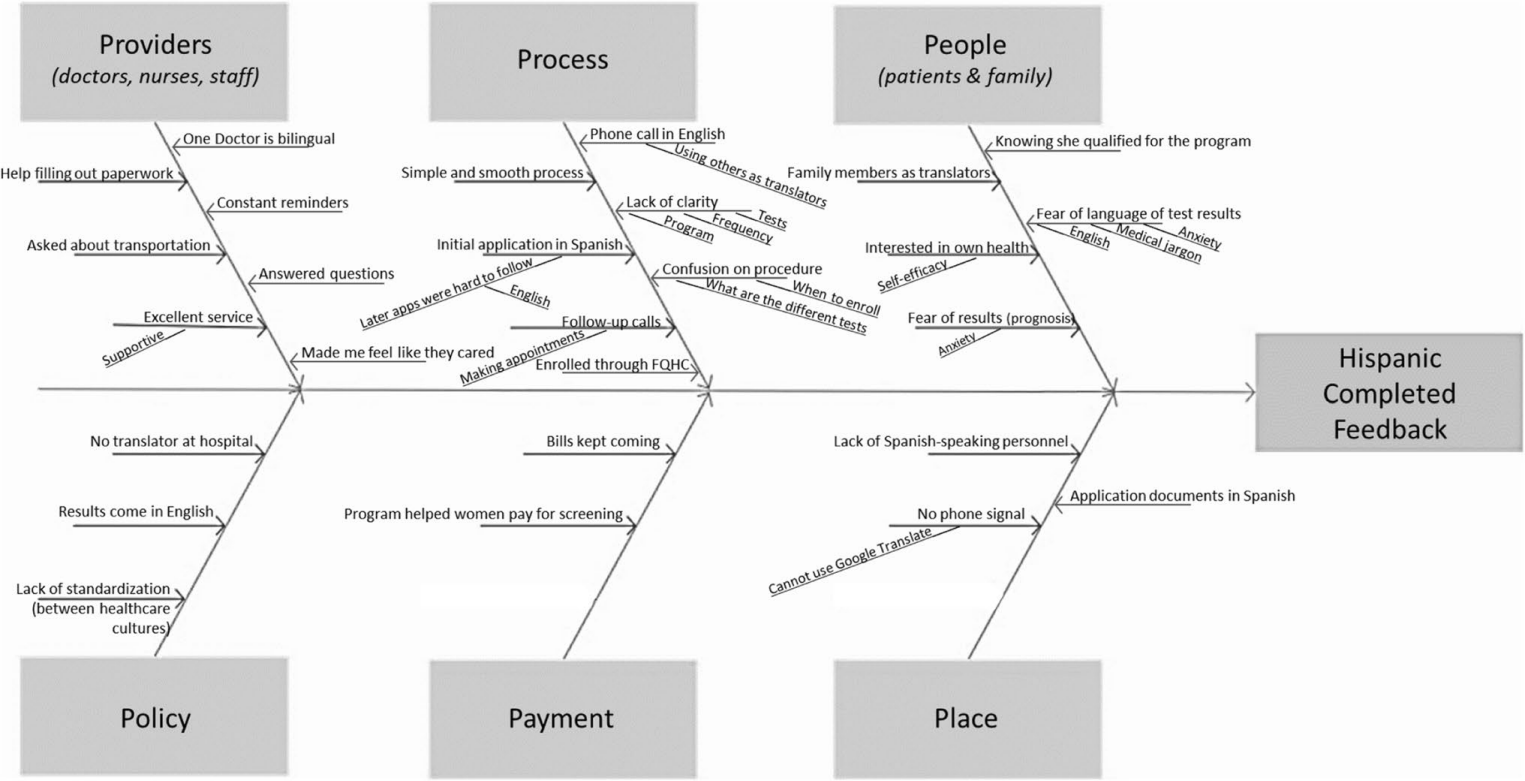
Fishbone diagram constructed from Hispanic women who completed screenings

They also told us that they received bills for the services in English that were confusing and difficult to understand (Fig. 3).

### Hispanic Women Who Did Not Complete Screenings

These women did not mention the providers’ offices or the program coordinator and did not understand the program. One woman was concerned that she was being enrolled in a research study and both women said they did not trust the program. These women expressed frustration at the forms and information presented in English and had confusion about the role of insurance in the process. One woman explained:

*First of all, I’m in a foreign country. So a lot of the processes, I don’t know. They told me that they could do a mammography study and that all I needed was to register and that they were going to contact me and they gave me a form. And this form was in English, but I could not read it in English because I am not fluent in English. I am not fluent in English. It is complicated for me not to have information in Spanish.*

**Fig. 4:**
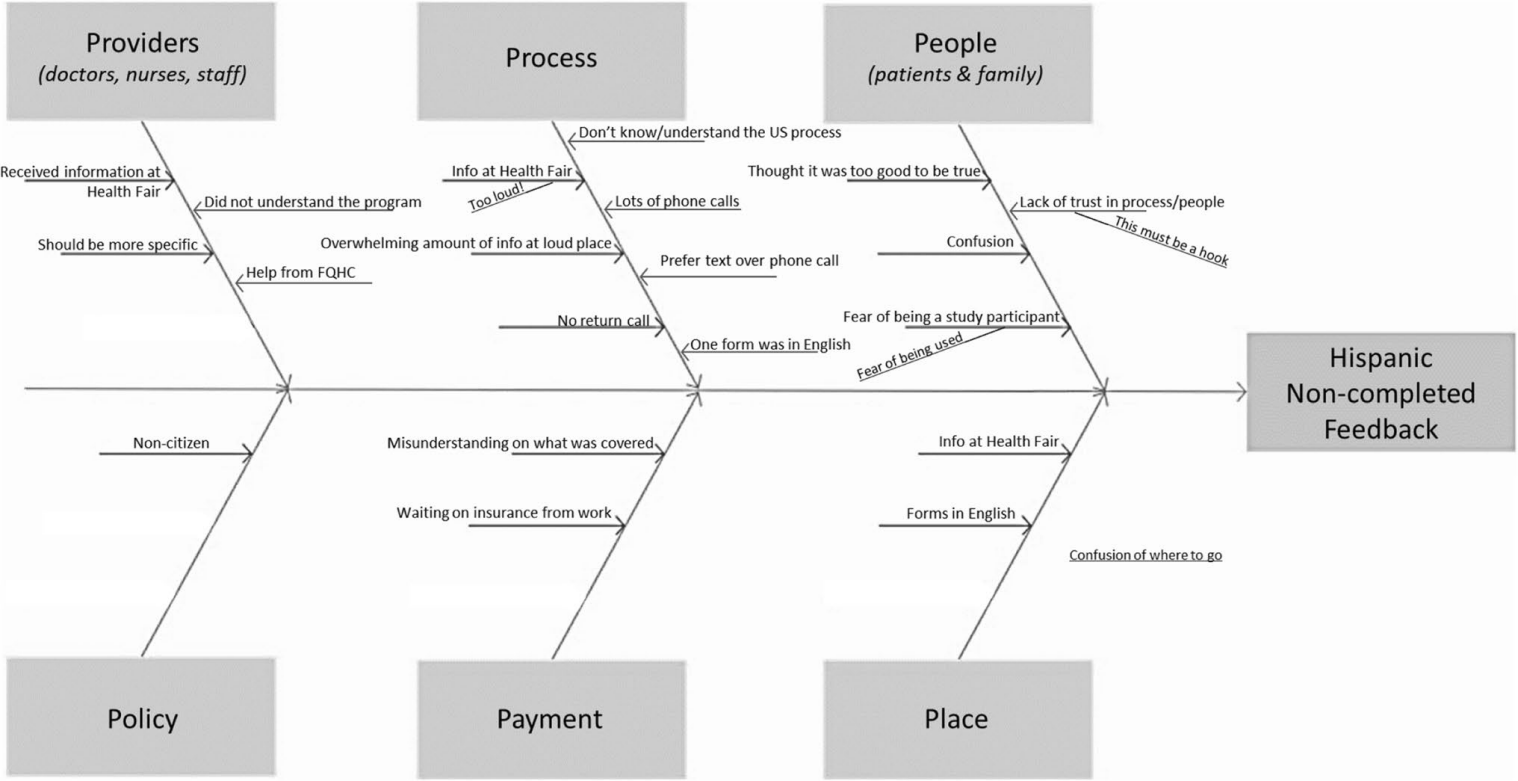
Fishbone diagram constructed from Hispanic women who did not complete screenings

Both women were worried about their status as non-US citizens and their eligibility for the program (Fig. 4).

## Discussion

We present findings from a community-based participatory research project to examining barriers and facilitators to breast and cervical cancer screenings in southwest Montana. We found that there are differences for non-Hispanic White women and Hispanic women due to language, the role of insurance, and the level of trust in the program. Researchers often divide perceived barriers to screening into two categories of structural barriers and personal barriers [29]. Structural barriers in other work include cost, [14, 30, 31] limited time off work, [31] transportation, [14, 32, 33] language barriers, [29, 33–35] and provider discrimination [30]. Personal barriers may include fear, [36, 37] embarrassment, [31] low health literacy, [36, 38] low levels of acculturation, [32, 39] and not wanting to be screened by a male physician [29, 31]. We found the barriers for Hispanic women were structural and systemic in nature and included language, limited health literacy, and difficulty with payment information. Hispanic women reported not understanding the structure of the program and confusion when bills inadvertently arrived. They also expressed mistrust in the program and fear about their documentation status. However, the non-Hispanic White women who did not complete the recommended screenings did not report structural barriers; they made the choice not to complete screenings for personal reasons.

Other researchers have found similar structural barriers for women of minoritized communities attempting to access healthcare screening services, especially in rural settings. For example, in interviews with providers, Maar and colleagues found health literacy and transportation to be barriers for First Nations women, [40] and a similar investigation among Hmong women by Fang and Baker found health literacy and language to be significant barriers [41]. In a meta-synthesis, Corcoran and Crowley note the existing barriers for Hispanic women, specifically, surrounded unfamiliarity with the healthcare system and language. They also note that confusion around cost of screenings prevented women from accessing available services [30]. These findings are echoed in our results. Additionally, the most common and consistent barrier to completing recommended screenings we found in our study was language. Language barriers influence access to care and have been noted in multiple studies of non-English speakers’ rates of cancer screening [29, 33–35]. Montana has not traditionally been a state where Hispanic persons migrate, and services in Spanish are only just being developed.

Both groups of women who completed recommended screenings reported anxiety about receiving potentially positive test results. In a review of the literature, Austin and colleagues identified fatalism to be a barrier to cancer screening among Hispanic women, stating that a fear of dying from cancer prevents many women from accessing available screenings [31]. Moreno and colleagues found that fatalism played a role in screening completion, but that the role was marginal, suggesting that systematic factors, including familiarity with the healthcare system, are more influential [42]. In contrast, Corcoran and Crowley found that Hispanic women view screening as a way to protect their health, [30] which is similar to our finding that a desire to know about one’s health was a facilitator to completing screenings. None of the women who did not complete screenings reported a fear of results, however.

Facilitators for screening included the role of helpful community members who were familiar with the program, bilingual staff, easy paperwork, and a smooth and simple process. Among the facilitators that were most salient in our findings was the role of other people who helped women access screenings. Both non-Hispanic White and Hispanic women noted that having assistance with scheduling appointments, having help with transportation, or receiving reminders from the program administrator were positive factors. The influence of other people has been capitalized on by many researchers and public health officials through the use of community health workers in promoting cancer screenings [43–46]. In Texas, for example, Shokar and colleagues report on a community health worker model called *De Casa en Casa* that provides culturally relevant education, navigation services, and a dedicated screening clinic to Hispanic women [47]. They report completed screening rates of nearly 67% among women who received the services. Implementation of targeted community health worker models may improve screening completion for both non-Hispanic White and Hispanic women.

Other facilitators included bilingual staff, easy enrollment paperwork, and a simple and smooth process. The Hispanic women in our study were most grateful to the bilingual clinic staff who were able to explain the program, coordinate the screening visits, and provide results in Spanish. Staff at providers’ offices are often the first contact for women eligible for NBCCEDP, and they play an integral role in the success of the program. Educating clinic staff in the procedures and creating a streamlined process to communicate eligibility to mammogram providers may be beneficial.

While our study has many strengths, it has limitations that must be addressed. The biggest limitation is the small sample size for the women who did not complete the NBCCEDP. We conducted multiple attempts to enroll women, including text messages, repeat phone calls, and involving a CAB member who had access to the women through her job at the local health clinic. The three principal investigators conducted a fishbone diagram to examine the potential reasons women did not engage in the research. We hypothesized that women who did not prioritize health screenings were not likely to prioritize participating in a research study. Another limitation was the reliance on the memories of women who had been enrolled in the program in the previous 12–18 months. We attempted to include women who had recently been enrolled, but their memories may not be fully accurate. Finally, we did not examine levels of acculturation in our study, but Coronado and colleagues found that Hispanic women with lower levels of acculturation reported more barriers to completing cancer screening when compared to Hispanic women with higher levels of acculturation or non-Hispanic White women [34]. Understanding the role of potential confounding factors including acculturation and health literacy would add to our findings.

Our research examined barriers and facilitators among non-Hispanic White women and Hispanic women for their enrollment in the NBCCEDP in Montana. We seek to understand potential barriers and facilitators for women as they attempt to complete the recommended cancer screenings. We also aimed to determine if the barriers and facilitators varied between non-Hispanic White women and Hispanic women. Barriers to completion of the NBCCEDP include confusion about health insurance, unexpected medical bills, and fear of a potentially positive cancer screening test result. The facilitators include support and reminders from program administrators and staff, an efficient enrollment process, and a desire to learn about their own health.

Despite overlap in the barriers and facilitators for Hispanic and non-Hispanic White women, our research concludes that Hispanic women face more systemic and structural barriers to NBCCEDP completion when compared to non-Hispanic Whites in Montana. Understanding and addressing the differences in barriers to completion of cancer screening programs between Hispanic women and non-Hispanic women are essential to decreasing the health disparities in the screening, development, and diagnosis of reproductive cancers in women. Additional research is needed to analyze and determine potential programs that could work to successfully address these differences in barriers.

## Data Availability

The authors confirm that the data supporting the findings of this study are available within the article and/or its supplementary materials.
